# Practice of breast self-examination and associated factors among female health professionals working in public hospitals of Harari regional state: Eastern Ethiopia multicenter study

**DOI:** 10.3389/fonc.2022.1002111

**Published:** 2022-11-21

**Authors:** Deribe Bekele Dechasa, Henock Asfaw, Lemesa Abdisa, Yadeta Dessie, Tilahun Bete, Addisu Sertsu, Ahmed Hiko, Teganu Balcha, Addis Eyeberu, Kabtamu Nigussie, Dawit Tamiru

**Affiliations:** ^1^ School of nursing and midwifery, College of Health and Medical Science, Haramaya University, Harar, Ethiopia; ^2^ School of Public Health, College of Health and Medical Science, Haramaya University, Harar, Ethiopia

**Keywords:** breast cancer, breast self-examination, practice, female, Ethiopia

## Abstract

Breast cancer is the most frequent malignancy among women in both developed and developing countries. Early detection of breast cancer using breast self-examination plays a substantial role in reducing its morbidity and mortality. Despite improved availability of health information the morbidity and mortality caused by breast cancer among females were increasing from time to time and there is no information regarding the practice of breast self-examination in our study area.

**Objective:**

This study was aimed at assessing breast self-examination practice and associated factors among female health professionals who were working in public hospitals of Harari Regional State in 2022.

**Methods:**

Institutional-based quantitative cross-sectional study design was used to assess the practice of breast self-examination and associated factors among female health professionals who have been working in a public hospital in Harari regional state from March 25 to April 05, 2022. The study subjects were selected using a simple random sampling technique and data were collected by a self-administered and structured questionnaire. The collected data was edited, cleaned, coded, and entered into Epi-Data version 3.1 software and analyzed using the statistical package for social science software version 20. Bivariable and multivariable logistic regression analysis was carried out to see the association between independent and dependent variables. Variable with P-value less than 0.05 at final model were regarded as statistically significant.

**Result:**

Among a total of 362 female health professionals, 171 (47.2%) respondents were ever practiced breast self-examination, 229(63.3%) had good knowledge of breast self-examination and 252(69.6%) had a favorable attitude toward breast self-examination. Work experience of ≥5 year (AOR = 2.51; 95% CI: 1.31–4.82), educational status of degree and above (AOR = 7.2; 95% CI: 3.82–10.58), good knowledge about breast self-examination (AOR = 3.4; 95% CI: 1.41–5.40) and favorable attitude toward breast self-examination (AOR = 3.1; 95% CI: 2.11–4.10) were significantly associated with breast self-examination practice.

**Conclusion:**

The finding of this study implies that the practice of breast self-examination among female health professionals is low. Work experience of ≥5 year, educational status of degree and above, having a good knowledge and favorable attitude toward breast self-examination were significantly associated with breast self-examination.

## Introduction

### Background

Breast cancer is the leading cause of mortality by cancer in developing countries among the women and it is the second leading cause of mortality by cancer next to the lung cancer among women in the developed countries (1). According to 2018 data on breast cancer estimation, about 626,679 women died from breast cancer in the world which accounted for a crude mortality rate of 13 per 100,000 women. Around 10, 000 Ethiopian women excluding those who were not reported since they were living in remote and rural areas and who seek traditional treatment have already developed breast cancer (2, 3).

According to the 2018 Global Cancer Incidence, Mortality, and Prevalence (GLOBOCAN) report, there were around 2.1 million newly diagnosed female breast cancer cases in 2018, accounting for nearly one-fourth of all cancer cases among women (2). Around 980,000 new cancer cases are expected to be diagnosed globally until 2040 (4).

Breast self-examination (BSE) is a breast cancer screening method in which a woman examines and feels her breasts for lumps, distortions, and swellings. BSE is a straightforward procedure that has the potential to save the lives of women. Every woman above the age of 20 is advised to have BSE once a month for 20 minutes (5). Breast self-examination is a low-cost, painless, simple, safe, and non-invasive process that does not require any specific materials or tools. It’s a crucial breast cancer detection method that only takes five minutes to implement (6).

Globally the practice of breast self-examination varies from country to country. The cross-sectional study conducted among female health professionals in Pakistan showed that 54% (7) of the study participant had ever practiced breast self-examination which is lower than the study conducted in Iran (66.4%) (8), Nigeria (94.6%) (9), and Western Ethiopia (77.1%) (10). Additionally, the study conducted among female health professionals in Dire Dawa revealed that 38.2% (11) of the study participants reported that they practiced breast self-examination.

However, due to a lack of awareness and expertise about breast self-examination, the majority of women do not perform it. The absence of signs and symptoms, fear, pain, lack of advice, forgetting the schedule, environmental variables, lack of cultural support, and lack of support from spouses were identified as factors that prevent women from performing breast self-examination (12–14). As a result breast cancer mortality rate is much higher in developing countries than in developed countries (2, 15–18). Even though many studies were conducted in the United States (US), Europe, and Africa but there are a few studies done in Ethiopia and there is no study conducted on eastern Ethiopian female health professionals in the Harari region. Hence, this study was aimed to assess the Practice of breast self-examination and associated factors among female health professionals in public hospitals from March 25 to April 05, 2022.

## Materials and methods

### Study area and period

The research was carried out in the Harari regional state. Harari regional state is one of Ethiopia’s eleven states, with a total population of 246,000 people, 124000 of them are men and the rest are females. The Harari region is estimated to be 333.94 square kilometers in size, with a population density of 595.9 persons per square kilometer. The distance between Addis Ababa and Harari is 526 kilometers to the southeast. Oromia regional state borders this region on the east, west, north, and south (19, 20).

Two of the public hospitals are Hiwot Fana Comprehensive and Specialized Hospital and Jogol hospital. They deliver multi-dimensional health services approximately for five million populations in the catchment area. Jogol Hospital is a regional referral hospital with 97 beds and 303 health professionals of which 147 are female health professionals. The study was conducted in two public hospitals in Harari (Hiwot Fana Comprehensive and Specialized Hospital and Jogol hospital). In both of the hospitals, there are 422 female health professionals. The study was conducted from March 25 to April 05, 2022.

### Study design and population

An institutional-based quantitative cross-sectional study design was employed. All-female health professionals who were working in the public hospitals of Harari regional state, eastern Ethiopia were the source population. The study participants were female health professionals who have been working in the hospitals for the last 6 months before the data collection period and who are available during the data collection period. Female health professionals who were on sick leave, annual leave, and maternal leave during the data collection period were excluded from the study.

### Sample size determination

The outcome variable and the factors that were significantly associated with the outcome variable were taken into account when determining the sample size for this study. The sample size for the first and second objectives was computed individually, and the one with the highest number was used for this study.

For the first specific objective, a single population proportion was used to determine the sample size of the study. For this study p=33.7% from the study on breast self-examination practice and associated factors among female health professionals in Western Ethiopia, (10). Therefore;


$$n=\;\frac{{\left(1.96\right)}^{2}\;0.337\;(1-0.337)}{0.05^{2}}\;=\;343$$

By adding 10% (34 of participants) for non -response rate gives; n=377.

The sample for the second specific objective is determined by using the Epi Info version 7 by considering the factors that were significantly associated with breast self-examination practice at (p=0.05), a two-sided confidence level of 95% and margin of error of 5%, and power=80 percent, and a ratio of exposed to unexposed of 1:1.To get enough sample size we used the factor that gave us a large sample size and accordingly sample size was 330 and 10% non-response rate was added. By adding 10% to 330 it gave us 363 which is less than 377. Since 377 is larger than 363, thus the final sample size was 377.

### Sampling procedure

The study was conducted in public hospitals in Harari Regional State. Both Hiwot Fana Comprehensive and Specialized Hospital and Jogol Hospital were included in this study, and the determined sample size was proportionately allocated to each hospital based on the total number of female health professionals of both public hospitals. The total number of female health professionals in both public hospitals was 422. The sampling frame of female health professionals was prepared and female health professionals were selected by using a simple random sampling method after proportional allocation was done for both hospitals.

**Figure 1:**
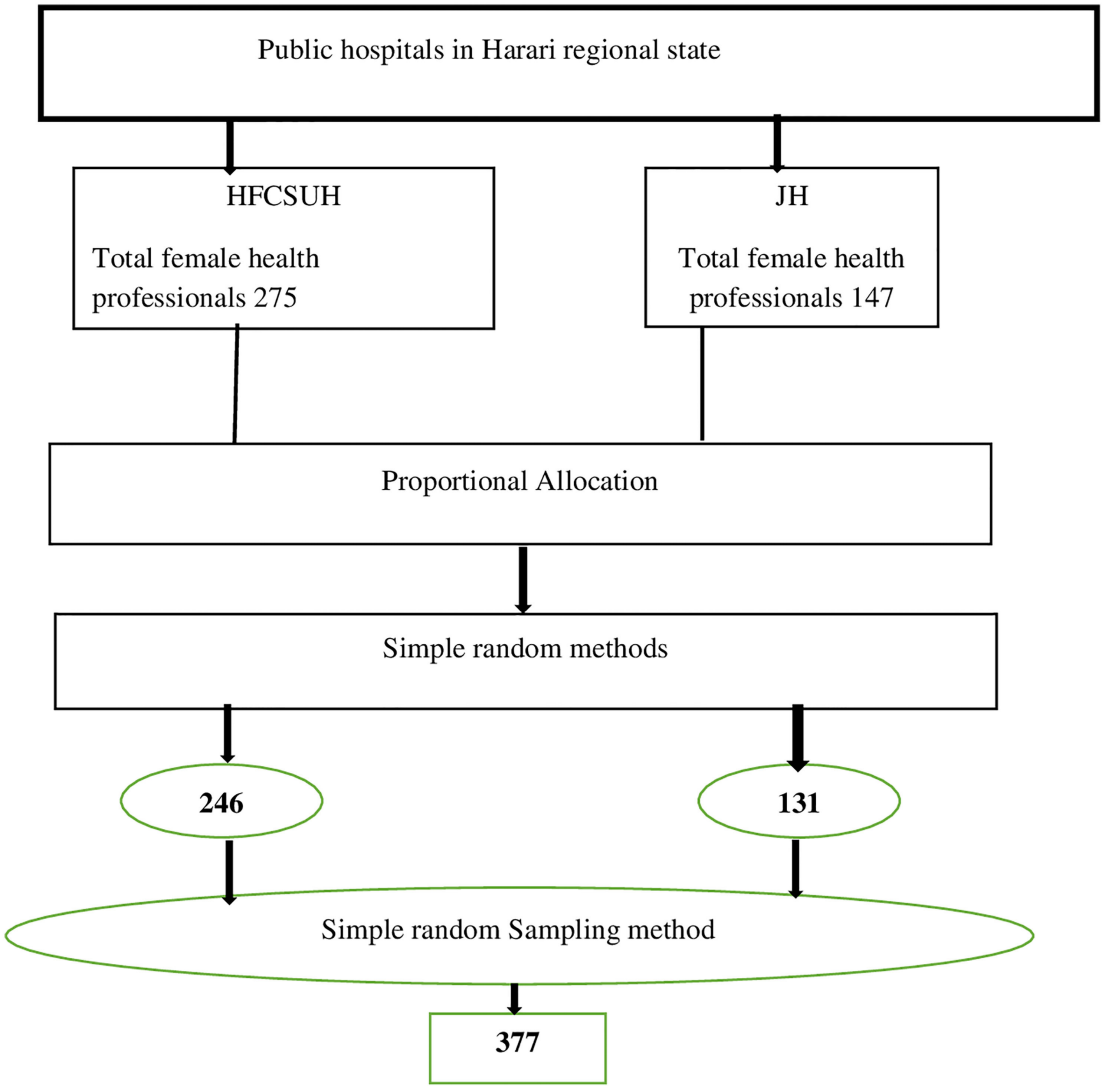
The schematic presentation of the sampling procedure for the study on the breast self examination practice and associated factors among female health professionals working in Harari regional state, 2022.

The total number of female health professionals from Hiwot Fana Comprehensive and Specialized Hospital was 275, hence the number of female health professionals proportionally allocated was: 275x377/422 = 246. The total number of female health professionals from Jogol Hospital was 147, hence the number of female health professionals proportionally allocated was: 147x377/422 = 131 as shown in below (**Figure 1**).

### Data collection tools and procedure

The data were collected by a structured questionnaire which was adapted and modified after reviewing and extracting from different pieces of literature developed for the same purpose (11). The tool has four sections: the first contains items for personal information or sociodemographic components, the second contains items for assessing female health professionals’ knowledge, the third contains items for assessing female health professionals’ attitude toward breast self-examination, and the fourth section contains items for determining breast self-examination practice.

Two hospitals from Harari regional state were identified for this study. Female health professionals were selected from each hospital and the total sample size was met by a simple random sampling method based on proportional allocation done for both hospitals. A structured and English version of the questionnaire was distributed to all female health professionals who have filled the consent by their will. Two BSc nurses were assigned per each hospital and from different hospitals other than the hospitals in which the study was conducted and one supervisor who is a BSc holder in Nursing was assigned per each hospital.

### Data quality control

The questionnaire was written in English and then translated into Amharic to ensure consistency. The participants in the study were given a brief orientation. A pre-test was undertaken on 5% of the total sample size in Haramaya hospital one week before data collection. Supervisors have reviewed every questionnaire for completeness and logical consistency, which was counter-checked by the principal investigator. There was cross-check of the total activity by principal investigators.

### Data processing and analysis

The collected data from each respondent was coded, edited, and entered into Epi Data version 3.1. Then data was exported to SPSS version 20 for analysis. The missing value was checked by running frequency and descriptive statistics. For bivariable analysis, all covariates were analyzed, and a covariate with a P-value less than 0.25 was considered for multivariable analysis. The 95% confidence interval was used to calculate the crude and adjusted odds ratios, which were used to assess the strength of the association between the outcome and independent variables. In the multivariable analysis, variables with a P-value less than 0.05 were considered significantly associated with the outcome variable. Hosmer and Lemeshow were used to check the logistic regression model’s fitness.

### Operational definition

#### Good knowledge

If participants replied greater or equal to the mean score on the knowledge evaluation questions, they were considered to have good knowledge (21).

#### Poor knowledge

If a participant scored less than the mean on the knowledge evaluation questions, they were considered to have poor knowledge (21).

#### Favorable attitudes

Participants who scored points equal to or greater than the mean score of breast self-examination related attitude questions as measured by the Likert scale (17, 22).

#### Unfavorable attitude

Participants who scored points less than the mean score of attitude questions (17, 22).

## Results

### Socio-demographic profile of the participants

From a total of 377 samples, 362 participants were included in the study, with a response rate of 96%. The highest percentage of the respondents, 162(44.8%), were in the age range of 20–29 years. Regarding marital status, more than half of 196(54.1%) of the respondents were single and 40.1% were married. Of 362 respondents, 178(49.2%) were Orthodox Christians, and nearly two-thirds, 348 (96.1%), and 356(98.3%) of respondents have reported that they did not have any family history of breast cancer and personal history of breast problem respectively. Among respondents who had a family history of breast cancer was 14(3.9%) as shown in below (**Table 1**).

**Table 1:** Socio-demographic characteristics of female health professionals who were working in public hospitals of Harari Regional State, Eastern Ethiopia, 2022 (n=362).

Characteristics	Category	Frequency	Percentage
Age	20-29	148	40.8%
	30-39	162	44.8
	≥40	52	14.4
Marital status	Single	196	54.1
	Married	145	40.1
	Divorced	21	5.8
Religion	Orthodox	178	49.2
	Muslim	144	39.8
	Protestant	40	11
Profession	Nurse	154	42.5
	Midwifery	84	23.2
	Pharmacy	71	19.6
	Laboratory	9	2.5
	Medical doctor	19	5.2
	Others	16	4.4
Educational level	Diploma	49	13.5
	Degree and above	313	86.5
Current unit of service	Inpatient service	203	56.1
	Outpatient service	159	43.9
Experience in years	<5	265	72.8
	≥5	97	26.2
Any family history of breast cancer	Yes	14	3.9
	No	348	96.1
If yes what is her relationship with you	Mother	3	21.4
	Aunt	5	25.7
	Sister	6	42.9
Personal history of a breast problem	Yes	6	1.7
	No	356	98.3

### Knowledge of female health professionals about breast self-examination

**Figure 2:**
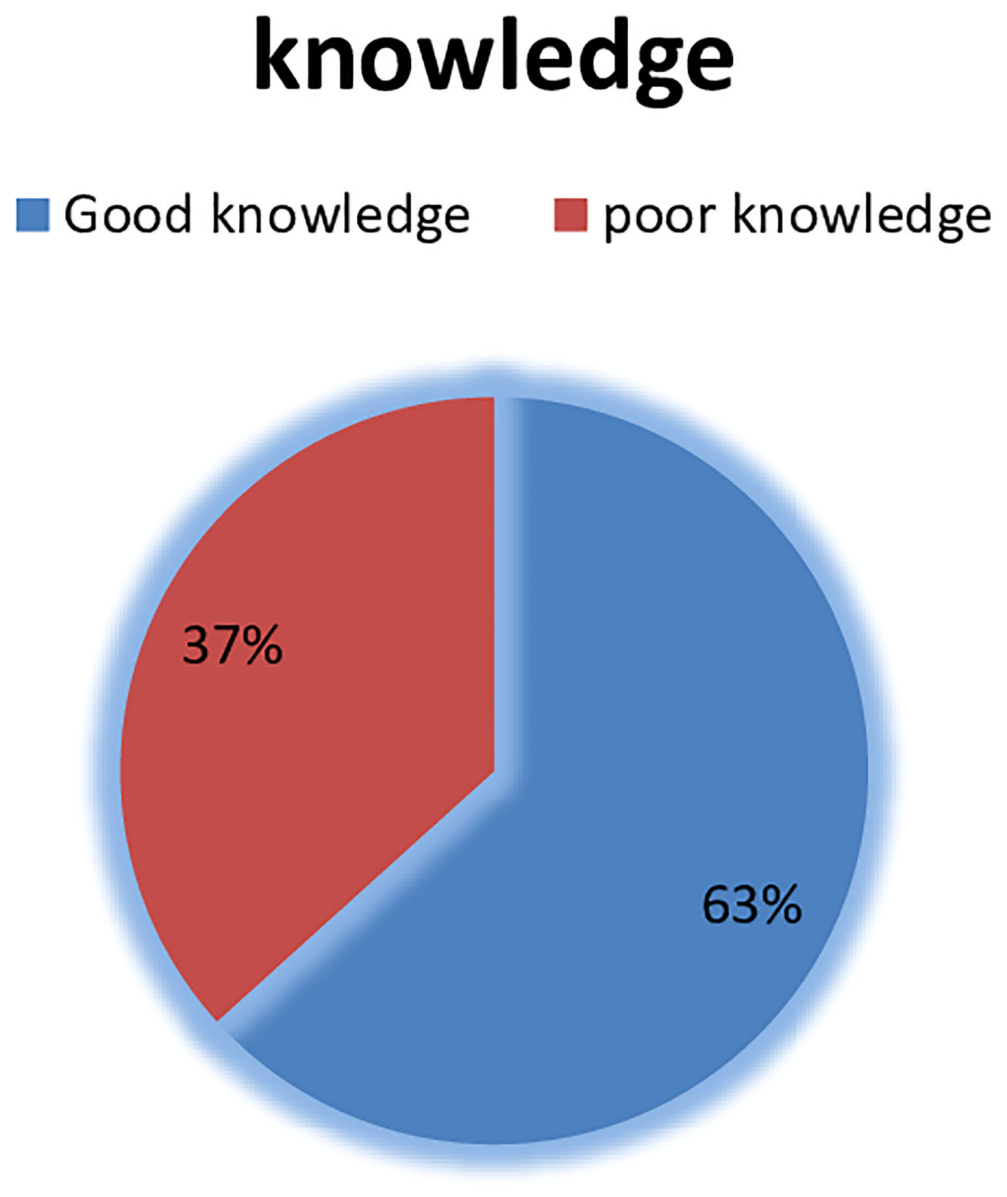
Knowledge of female health professionals who were working in public Hospitals of Harari regional state, Eastern Ethiopia, 2022.

Among female health professionals that participated in this study, 350(96.7%) of them reported that breast cancer affects more females than males. Early identification of breast cancer increases the likelihood of survival, according to nearly three-quarters of the survey participants, 338 (93.4%) and 318 (87.8%) reported that breast cancer is treatable if it is detected and diagnosed at an early stage. Among the prepared questions, the participants who answered the mean and above were considered to have good knowledge and those participants who had answered below mean were considered to have poor knowledge. Based on this, from 362 participants 229(63.3%) had good knowledge of breast self-examination as shown in below (**Figure 2**).

### Attitude of respondents regarding breast self-examination

From the total of 362 study participants, 252(69.6%) had a favorable attitude toward breast self-examination as shown in below (**Table 2**).

**Table 2:** Attitude of female health professionals who were working in public Hospitals of Harari regional state, Eastern Ethiopia, 2022 (n=362).

Variables	Category	Frequency	Percent (%)
Practicing BSE regularly and correctly is important to detect the disease at earlier stage	Strongly agree	129	35.6
	Agree	205	56.6
	Neutral	28	7.7
	Disagree	_	_
	Strongly Disagree	_	_
By detecting breast cancer through BSE, I can improve my chance of survival	Strongly agree	226	62.5
	Agree	70	19.2
	Neutral	11	3.1
	Disagree	43	12
	Strongly Disagree	12	3.2
I believe it is very useful to examine my breasts regularly	Strongly agree	130	35.9
	Agree	203	56.1
	Neutral	23	6.4
	Disagree	40	11.2
	Strongly Disagree	6	1.7
It is not difficult for me to remember to do regular BSE every month	Strongly agree	43	11.9
	Agree	200	55.2
	Neutral	40	11
	Disagree	65	18
	Strongly Disagree	14	3.9
**Attitude**	Unfavorable attitude	110	30.39
	Favorable attitude	252	69.61

### Female health professional’s breast self-examination practice

The finding of this study revealed that 171(47.2%) of the participants ever practiced breast self-examination; of them, 19.9% practiced regularly. Among the study participants who practiced breast self-examination, 55(15.2%) of the study participants performed BSE two to three days after cessation of menstruation. More than two-thirds of 107(29.6%%) of the study participants had started practicing BSE before the age of 25years. More than one-third, 83(46.9%) of the study participants performed breast self-examination less than five times in the last six months as shown in below (**Table 3**).

**Table 3:** BSE practice of female health professionals who were working in public Hospitals of Harari regional state, Eastern Ethiopia, 2022 (n=362).

Variable	Category	Frequency	Percentage (%)
Ever performed BSE?	Yes	171	47.2
	No	191	52.8
How often do you practice BSE (n=171)	On a weekly basis	14	3.9
	Once every month	72	19.9
	Every three months	48	13.3
	Rarely	37	10.2
Age started practicing BSE (n=171)	25 years or less	107	29.6
	Between the ages of 25 and 30,	64	17.7
	More than 30 years of age	6	1.7
When do you perform BSE? (n=171)	2 to 3 days following the last menstrual period	55	15.2
	Each month’s regular days	47	13
	A few days before menstruation	22	6.1
	When it comes to mind	53	14.6
How many times in the last 6 months did you perform BSE? (n=171)	five to six times	43	11.9
	Fewer than five times	83	22.9
	Didn’t perform	51	14

### Factors associated with female health professionals’ breast self-examination practice

Based on the conducted bivariable analysis, profession of the participant, unit of service at which they are giving service, work experience in a year, educational status, knowledge of the participants, and the attitude of the participants had associated with female health professionals’ breast self-examination practice. After a multivariable logistic regression analysis was done, female health professionals who had a work experience of ≥5 year, educational status of degree and above, good knowledge about breast self-examination, and favorable attitude toward breast self-examination were significantly associated with breast self-examination practice.

Female health professionals who had a work experience of ≥5 year were 2.5 times more likely to perform breast self-examination than those who had a work experience of< 5 years (AOR = 2.51; 95% CI: 1.31–4.82). Those female health professionals who had educational status of degree and above were 7.2 times more likely to perform breast self-examination practice than diploma holders **(**AOR = 7.2; 95% CI: 3.82–10.58**)**.

Female health professionals who had good knowledge about breast self-examination were 3.4 times more likely to examine their breasts than female health professionals who had poor knowledge about breast self-examination **(**AOR = 3.4; 95% CI: 1.41–5.40**).**

Attitude towards breast self-examination is another factor which is significantly associated factor to breast self-examination practice. Those female health professionals who had a favorable attitude towards breast self-examination were three times more likely to practice breast self-examination than those who had an unfavorable attitude **(**AOR = 3.1; 95% CI: 2.11–4.10**)** as shown in below (**Table 4**).

**Table 4:** Factors associated with BSE practice of female health professionals who were working in public Hospitals of Harari regional state, Eastern Ethiopia, 2022 (n=362).

Independent Variables	Category	Practice breast self-examination		COR (95% CI)	AOR (95% CI)
		Yes	No		
Profession	Nurse	72	82	0.26 (1.01-4-4.7)	0.55 (0.14-2.2)
	Midwifery	42	42	0.3 (0.85-4.35)	0.48 (0.12-1.9)
	Pharmacy	20	51	0.12 (2.1-11.7)	1.6 (0.4-6.5)
	Laboratory	14	4	1.1 (0.15-2.03)	0.22 (0.04-1.2
	Medical doctor	27	8	1	1
Current unit of service	Inpatient	81	122	0.51 (1.3-2.99)	1.3 (0.76-2.16)
	Outpatient	90	69	1	1
Work experience in year	<5 year	111	154	1	1
	≥5 year	60	37	2.25 (1.22-3.28)	2.51 (1.31-4.82)*
Educational status	Diploma	8	41	1	1
	Degree & above	163	150	5.60 (3.82-7.38)	7.20 (3.82-10.58)*
Knowledge about BSE	Poor knowledge	53	80	1	1
	Good knowledge	121	108	1.70 (0.86-2.54)	3.40 (1.41-5.40)**
Attitude toward BSE	Unfavorable attitude	30	80	1	1
	Favorable attitude	91	161	1.51 (0.95-2.07)	3.1 (2.11-4.10)**

*p < 0.05, and **p < 0.01.

Hosmer and Lemshow= 0.67.

## Discussion

The finding of this study revealed that 47.2% with (95% CI 40.6%- 53.8%) of the participants ever practiced breast self-examination. This finding is almost consistent with the study conducted in Debra Tabor (42.1%, Northern Ethiopia (21), and Nigeria (51%) (23). However the finding of this study is lower than the study conducted in Western Ethiopia (77%) (10), Gambella Ethiopia (61.5) (24), Iran(66.4%) (8), Nigeria(94.6%) (9), and Pakistan(54%) (7). The difference observed could be due to the difference in socio-economic and demographic characteristics of participants. Difference in sample size and study design is also contributing factors for this discrepancy. The studies from Iran and Pakistan were conducted among only 119 and 150 participants respectively and regarding difference in design, study from Iran used cluster sampling technique and study from Pakistan used convenience sampling technique, whereas in this study we used 362 samples by using simple random sampling.

But the result of this study is higher than the studies conducted in Dire Dawa, Eastern Ethiopia (38.2%) (11), West Shoa Zone, Western Ethiopia (32.6%) (25) and Pakistan (15.6%) (7). The possible difference could be due to the difference in time limit to assess breast self-examination practice. In this study we used ever practice of breast self-examination but in Diredawa study they used practice of BSE in one month base (11), and the studies from West Shoa Zone and Pakistan used the magnitude of BSE in regular base (25) (7).

Female health professionals who had a work experience of ≥5 year were 2.5 times more likely to perform BSE than those who had a work experience of< 5 years (AOR = 2.51; 95% CI: 1.31–4.82). This finding is consistent with the study findings from Dire Dawa, Eastern Ethiopia (11) and West Shoa Zone (25). The possible explanation is that female health professional with more than five years of work experience are more likely to receive information about BSE practice due to increased exposure to different health related practices compared with those with less than five years of work experience.

Female health professionals who had educational status of degree and above were 7.2 times more likely to perform breast self-examination practice than diploma holders. This finding is consistent with the study findings from Dire Dawa (11) and West Shoa Zone (25). The possible explanation is that those with educational level of degree and above might have better knowledge and skill than diploma holders to perform BSE.

The practice of BSE was significantly associated with having good knowledge about BSE practice of the respondents. Those with good knowledge about BSE practice were 3.4 times more likely to perform BSE practice as compared to those who have poor knowledge. This finding is comparable with the study finding of Dire Dawa (11), Western Shoa Zone (25), Nigeria (9), and Iran (8). The possible explanation is that knowledge about impact of breast cancer and having information on diagnostic methods of breast cancer enables them to perform BSE.

Female health professionals who had a favorable attitude toward breast self-examination were three times more likely to practice breast self-examination than those who had an unfavorable attitude. This report is consistent with the study conducted in Dire Dawa (11) and Western Ethiopia (10). The possible explanation is that if they have enough information about merits of BSE, it will help them to have good attitude towards BSE practice.

## Conclusions

The finding of this study implies that the practice of breast self-examination among female health professionals who have been working in public hospitals of Harari Regional State, Eastern Ethiopia is low. Female health professionals who had a work experience of ≥5 year, educational status of degree and above, having a good knowledge and favorable attitude toward breast self-examination were significantly associated with breast self-examination practice. Short term training on breast self-examination should be better which is organized by the hospital administrators.

## Strength and limitations of the study

This study provides important insights for health institutions and policymakers who are interested in improving community awareness of breast self-examination in general. However, as it was a cross-sectional study, causality cannot be determined.

## Data availability statement

The raw data supporting the conclusions of this article will be made available by the authors, without undue reservation.

## Ethics statement

The studies involving human participants were reviewed and approved by Haramaya University college of health and medical science. The patients/participants provided their written informed consent to participate in this study.

## Author contributions

DD, LA, HA, YD, TB, KN, AE, TB AH, DT and AS were participated in inception of idea, proposal development, data collection, analysis, and final write up. HA and DD and have participated on write up of the manuscript. All authors contributed to the article and approved the submitted version.

## Acknowledgments

We would like to express our deep gratitude to our data collectors for their admirable commitment and contribution. Also, our appreciation goes to study participants who willingly contributed to this study.

## Conflict of interest

The authors declare that the research was conducted in the absence of any commercial or financial relationships that could be construed as a potential conflict of interest.

## Publisher’s note

All claims expressed in this article are solely those of the authors and do not necessarily represent those of their affiliated organizations, or those of the publisher, the editors and the reviewers. Any product that may be evaluated in this article, or claim that may be made by its manufacturer, is not guaranteed or endorsed by the publisher.

## Abbreviations

BSE, Breast Self-Examination; CBE, Clinical Breast Examination; CSA, Central Statistics Authority; FMOH, Federal Ministry of Health; GLOBOCAN, Global Cancer Incidence; Mortality and Prevalence; HFCSH, Hiwot Fana Comprehensive and Specialized University Hospital; JH, Jogol Hospital; OPD, Out Patient Department; SPSS, Statically package for social science software; US, United State.
